# Schottky barrier memory based on heterojunction bandgap engineering for high-density and low-power retention

**DOI:** 10.1186/s11671-024-04106-5

**Published:** 2024-10-07

**Authors:** Hyangwoo Kim, Yijoon Kim, Kyounghwan Oh, Ju Hong Park, Chang-Ki Baek

**Affiliations:** 1 Future IT Innovation Laboratory, Pohang University of Science and Technology (POSTECH), Pohang, 37673 South Korea; 2 Department of Convergence IT Engineering, Pohang University of Science and Technology (POSTECH), Pohang, 37673 South Korea; 3 Department of Electrical Engineering, Pohang University of Science and Technology (POSTECH), Pohang, 37673 South Korea

**Keywords:** Capacitorless DRAM, Schottky junction, Heterojunction, Bandgap engineering, Holding voltage

## Abstract

Dynamic random-access memory (DRAM) has been scaled down to meet high-density, high-speed, and low-power memory requirements. However, conventional DRAM has limitations in achieving memory reliability, especially sufficient capacitance to distinguish memory states. While there have been attempts to enhance capacitor technology, these solutions increase manufacturing cost and complexity. Additionally, Silicon-based capacitorless memories have been reported, but they still suffer from serious difficulties regarding reliability and power consumption. Here, we propose a novel Schottky barrier memory (SBRAM), which is free of the complex capacitor structure and features a heterojunction based on bandgap engineering. SBRAM can be configured as vertical cross-point arrays, which enables high-density integration with a 4F^2^ footprint. In particular, the Schottky junction significantly reduces the reverse leakage current, preventing sneak current paths that cause leakage currents and readout errors during array operation. Moreover, the heterojunction physically divides the storage region into two regions, resulting in three distinct resistive states and inducing a gradual current slope to ensure sufficient holding margin. These states are determined by the holding voltage (*V*_hold_) applied to the programmed device. When the *V*_hold_ is 1.1 V, the programmed state can be maintained with an exceptionally low current of 35.7 fA without a refresh operation.

## Introduction

The ongoing shrinking trend in dynamic random-access memory (DRAM) aims to achieve high-capacity, fast, and energy-efficient memory solutions [1–3]. However, traditional one-transistor and one-capacitor DRAM (1T-1C DRAM) struggles to maintain memory reliability, primarily in preserving sufficient capacitance to distinguish individual memory states [4]. Despite attempts to improve capacitor technology by incorporating high-k dielectric materials and three-dimensional structures, these approaches result in increased production costs and added complexity [5–7].

In order to overcome these limitations, extensive research has been conducted on silicon-based high-density memories without a capacitor structure due to its high compatibility with conventional Silicon CMOS processes and high-speed operations [8–12]. Among them, bistable resistor (biristor) and thyristor RAM (TRAM) have received much attention, with many studies being focused on their primary memory mechanisms [13–16]. The biristor memory offers significant advantages in achieving highly integrated arrays due to its straightforward design8. However, the gate-less symmetrical structure introduces inherent sneak leakage currents, leading to increased readout errors and power consumption. In addition, this limitation imposes constraints on the maximum attainable array size [17]. The TRAM has the advantage of maintaining data with low standby power through a holding voltage without the need for the refresh operation [11, 12]. However, a notable concern arises from the steep current slope near the region where the holding voltage is determined [11, 12]. In the memory array configurations, a consistently low standby current is essential over a wide voltage range, considering device variations and operating voltage margins. The narrow holding margin in the TRAM ultimately leads to high standby power consumption. Silicon-based capacitorless memory devices have been extensively explored from various perspectives, but currently reported devices have significant limitations and are unsuitable for use as next-generation memory devices.

In this work, we have proposed a novel Schottky barrier memory (SBRAM) that uses heterojunction bandgap engineering for reliable and ultra-low power operations. Our device was designed with a straightforward configuration and high compatibility with the standard CMOS process. This design feature facilitates high-density integration with cost-effective pathways. The Schottky barrier at the anode electrode was demonstrated to prevent the sneak current, thereby increasing the reverse resistance in the programmed state. Additionally, the hysteresis curve characterized by three distinct current levels, was analyzed in terms of the role of two storage regions divided by heterojunction bandgap engineering. Therefore, the optimal holding voltage (*V*_hold_) was determined to ensure continuous and low-power retention characteristics.

## Results and discussion

A. Device structure and simulation

**Fig. 1:**
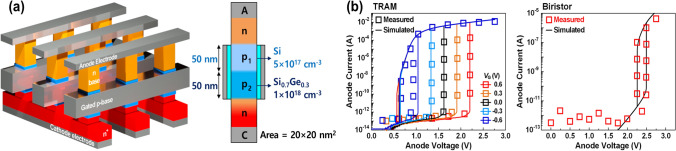
**a** Schematic diagram of SBRAM configured as a cross-point vertical array and cross section of unit cell composed of physical *n*–*p*_1_–*p*_2_–*n*^+^ layers. **b** Calibration simulation results (line) for hysteresis characteristics using experimental data (symbol) obtained from TRAM and biristor, both of which are Si-based floating memories

Figure 1a shows a schematic diagram of SBRAM in the form of a cross-point vertical array, representing one of the possible candidate array configurations, along with a cross-sectional view of the unit cell. Our device features a Si nanowire structure consisting of physical *n*–*p*_1_–*p*_2_–*n*^+^ layers, with the *p*_2_ base layer exceptionally made of silicon–germanium (SiGe) material. The germanium content (*x* in Si_1−x_Ge_x_) is set to 0.3 to minimize lattice mismatch and enable the deposition of a dislocation-free layer, which have high compatible with the standard CMOS process [18–21]. The channel area of the vertical nanowire is 20 × 20 nm^2^ for high-density array configuration. The gate electrode is titanium nitride (TiN) with a work function of 4.6 eV, a material commonly used in MOS devices [22]. The anode electrode is made of a Schottky metal with a Schottky barrier height of 0.5 eV, and is designed to prevent the reverse influx of carriers. The doping level of the *n*^+^ region is 10^20^ cm^−3^. The doping level of the *n*-base is 10^18^ cm^−3^, which prevents Schottky tunneling and ensures the sufficient impact ionization effect. The doping level and length of the *p*_1_-base are 5 × 10^17^ cm^−3^ and 50 nm, aiming to achieve low standby power, as will be discussed later. In the *p*_2_-base, the doping and length are10^18^ cm^−3^ and 50 nm, considering the margin for charge storage.

The SBRAM cell was simulated using Sentaurus technology computer-aided design (TCAD) [23]. Figure 1b shows the calibration results using hysteresis experimental data from TRAM and biristor—both Si-based floating body memories [24, 25]. This ensures the reliability of our simulation data. The calibration of TRAM considers both the energy barrier height and the resulting hysteresis window, both of which highly dependent on various gate biases [24]. For biristor calibration, the generation of charges through impact ionization and the resulting effects on the floating body is emphasized [25]. The physical parameters of recombination model were adjusted, and interface Shockley–Read–Hall (SRH) model was also adopted to reflect the data retention characteristics of real memory devices, which are significantly affected by junction and interfacial defects [26, 27]. Specifically, the interface defect density at the Si/SiGe interface was established using the measured data that are comparable to our design parameters [28]. A general drift–diffusion transport model, applied with Fermi–Dirac distribution, was used. The Philips unified mobility Model was applied to account for carrier scattering and doping-dependent mobility degradation [29]. The Oldslotboom bandgap narrowing model was adopted to consider the bandgap reduction in the heavily doped region [30]. An Avalanche generation model was used to calculate the generation of storage carriers through the impact ionization effect [31]. The voltage pulse width of all operations except the erase operation was set to 2 ns, which specifically surpasses the access speed of state-of-the-art 1T-1C DRAM memory [32]. The erase voltage pulse width was extended to 16 ns to reliable erasure of stored data.

B. Basic operational characteristics

**Fig. 2:**
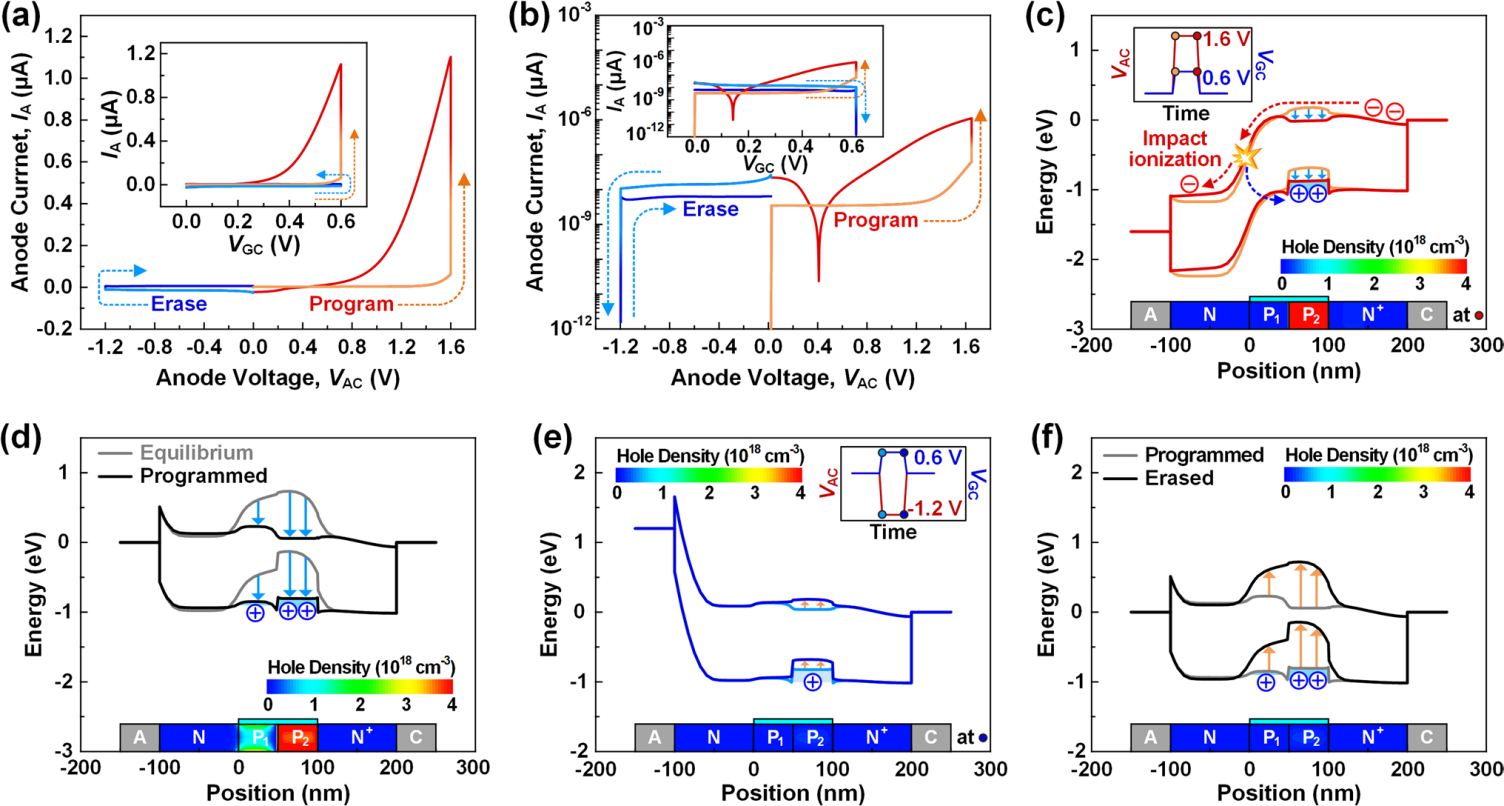
Asymmetric *I*_A _− *V*_AC_ and *I*_A _− *V*_GC_ (inset) hysteresis characteristics in the program and erase operations. **a** Linear scale. **b** Log scale. **c**–**f** Energy band diagrams and hole densities according to program and erase steps. **c** Latch-up process at *V*_AC_ = 1.6 V and *V*_GC_ = 0.6 V. **d** Comparison of equilibrium and programmed states at *V*_AC_ = 0.0 V and *V*_GC_ = 0.0 V. **e** Erasing process of stored holes at *V*_AC_ =  − 1.2 V and *V*_GC_ = 0.6 V. (**f**) Comparison of programmed and the erased states at *V*_AC_ = 0.0 V and *V*_GC_ = 0.0 V

Figure 2a and b show the asymmetric hysteresis characteristics of anode current-anode voltage (*I*_A _− *V*_AC_) and anode current-gate voltage (*I*_A _− *V*_GC_) in the program and erase operations of SBRAM. The voltage pulses with *V*_AC_ = 1.6 V and *V*_GC_ = 0.6 V are applied for the program operation. The program voltages were set to ensure stable hole generation and low power consumption. Then, latch-up occurs and *I*_A_ increases sharply to 1.1 μA. Then, a wide counterclockwise hysteresis loop is formed, indicating an increase in the potential of the *p*-base. Subsequently, voltage pulses with *V*_AC_ = − 1.2 V and *V*_GC_ = 0.6 V are applied for the erase operation. The erase voltages were set to achieve a stable erase operation. The device has the reverse *I*_A_ of − 1.6 pA. Unlike the program operation, no hysteresis loop is formed because the Schottky barrier prevents latch-up under reverse bias. The *I*_A_ level returns to its near initial state, which means the increased potential decreases.

The energy band diagrams and hole densities are extracted during the program and erase operation steps to closely analyze the changes in the base potential resulting from the memory operations as shown in Fig. 2c–f. Firstly, Fig. 2c shows the program latch-up process. When the *V*_GC_ increases to 0.6 V, the energy barrier height of the *p*-base decreases. When the *V*_AC_ of 1.6 V is applied simultaneously, electrons from the *n*^+^ region flow into the base region over the decreased energy barrier height. The injected electrons cause high-speed impact ionization in the high electric field area of the *n*-*p*_1_ junction, which generates electron–hole pairs. While maintaining the program voltages, the generated holes mainly accumulate in the *p*_2_-base composed of SiGe with a valence band offset. The accumulation of holes increases the potential of the *p*-base, facilitating the influx of electrons that lead to impact ionization from the *n*^+^ region to the base region. This sequence of processes activates the positive feedback, resulting in the program latch-up. As a result, the device transitions from a high-resistance state (*HRS*) to a low-resistance state (*LRS*). Figure 2d shows a comparison between the energy band diagrams of the equilibrium and programmed states. In the equilibrium state, the device shows a high energy band height in the *p*-base, indicating the *HRS*. Conversely, in the programmed state, the device shows a low energy band height in the *p*-base, indicating the transition to the *LRS*. This is because the generated holes by the program operation are maintained for a certain period, thereby increasing the potential of the *p*-base.

Next, Fig. 2e shows the erasing process of the stored holes in the program operation. When the *V*_GC_ increases to 0.6 V, an inversion layer is formed in the *p*-base. The stored holes are depleted as they recombine with electrons in the inversion layer. However, the erasing method, where only *V*_GC_ increases without adjusting *V*_AC_, may cause a problem in that electrons from the inversion layer flow into the n region, thus reducing the built-in potential. To preserve the built-in potential of the floating *n*-base, the *V*_AC_ should be decreased to − 1.2 V. Figure 2f shows the comparison between programmed and erased states with energy band diagrams. In the erased state, the hole density of the *p*-base returns to the equilibrium state, and thus the potential decreases. The increased energy band level, resulting from the reduced potential, indicates the transition of the device from *LRS* to *HRS*. What is important to note about this memory operation is that the Schottky barrier blocks the thermionic emission of electrons that cause the impact ionization in reverse bias. This blocked reverse current suppresses the generation of excess holes and thereby ensures the reliability of the erase operation. Additionally, symmetric devices without a Schottky junction have high reverse leakage current. These leakages can form sneak current paths between adjacent cells due to the high bidirectional conductivity. This results in increased read errors and power consumption and limits the maximum array size. In contrast, our unidirectional conduction characteristics can block the sneak current paths during cross-point array operations.

C. Bandgap engineering for low standby power consumption

**Fig. 3:**
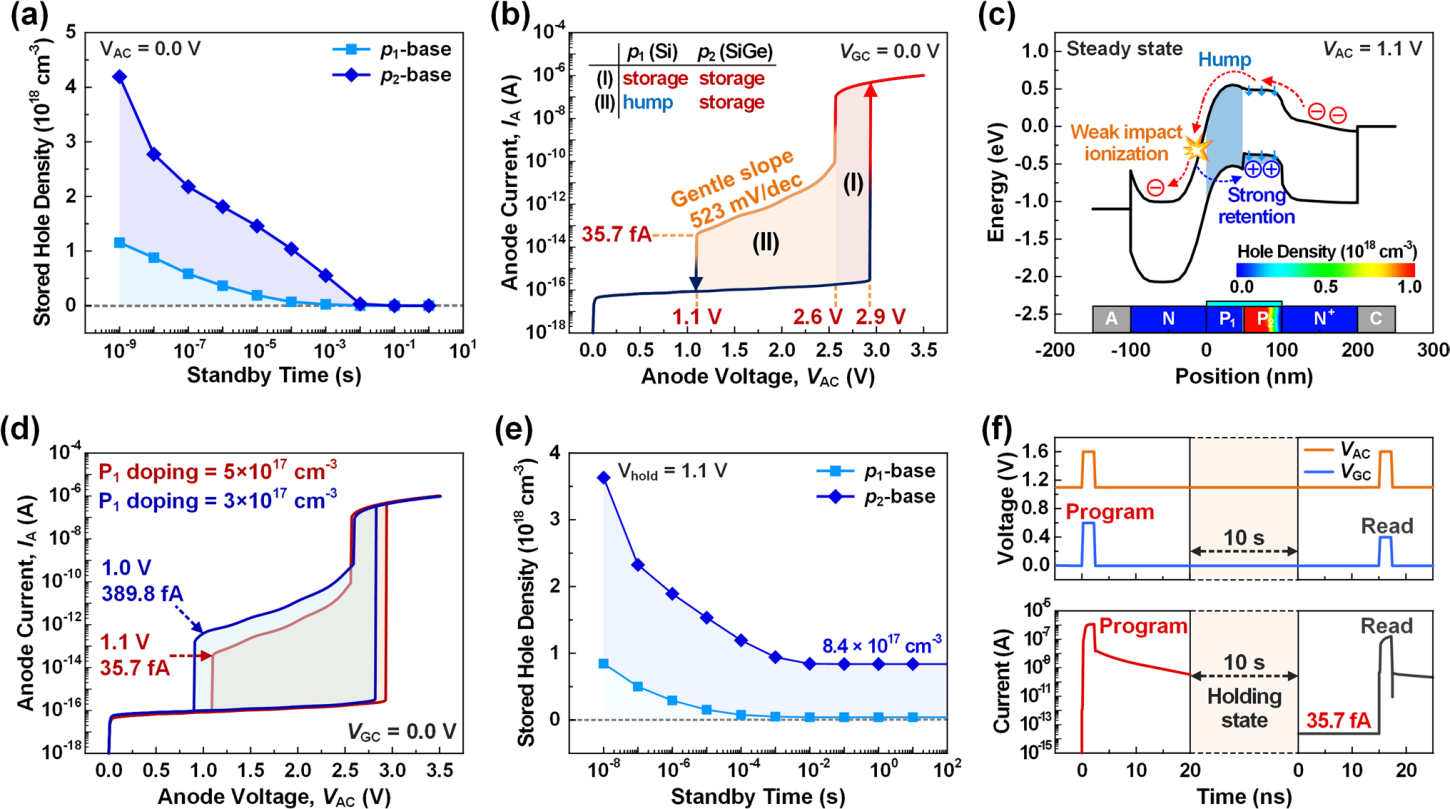
**a** Densities of the stored holes in the *p*_1_-base and the *p*_2_-base as a function of the standby time. **b** Quasi-static *I*_A _− *V*_AC_ hysteresis at *V*_GC_ = 0.0 V. The *LRS* is shown by dividing into (I) and (II) by the roles of the *p*_1_-and *p*_2_-base regions partitioned by hetero-junction. **c** Energy band diagram with *V*_AC_ = 1.1 V at the steady *LRS*, in which the weak impact ionization and strong retention regions are separated by the hump. **d** Comparison of quasi-static hysteresis when the *p*_1_-base doping is 5 × 10^17^ cm^−3^ and 3 × 10^17^ cm^−3^. **e** Stored hole densities in the *p*_1_-base and *p*_2_-base as a function of standby time when the *V*_hold_ of 1.1 V is applied. **f** The read voltages and current in the steady *LRS* when the *V*_hold_ is applied.

Figure 3a shows the stored hole density as a function of the standby time after programming. Although the *p*_2_-base has better retention characteristics than the *p*_1_-base due to its valence band offset, which effectively suppresses hole diffusion, the stored holes completely disappear after a standby time of approximately 10 ms. As such, capacitor-less floating body memories have poor retention characteristics compared to conventional 1T-1C DRAM, so more frequent data refresh operations may increase latency during data retrieval and consume additional power in most floating body memories [8–12]. Thus, it is necessary to apply a holding voltage (*V*_hold_) to ensure continuous retention characteristics by compensating for the loss of stored holes without the refresh operations. Figure 3b shows the quasi-static *I*_A_ − *V*_AC_ hysteresis curve for a grounded gate, i.e. *V*_GC_ = 0.0 V, to determine the minimum *V*_hold_ that can maintain the stored holes with minimal standby power consumption. When the *V*_GC_ is grounded, latch-up is closely related to the open-base breakdown of the bipolar junction transistor (BJT) [33]. The memory state according to latch-up can be explained by the amplification of *I*_A_ through two factors: current gain (*β*) and multiplication factor (*M*), which is expressed in the following Eq. (1):

1 $$I_{A} = \frac{M \cdot \beta }{{1 - (M - 1) \cdot \beta }} \, I_{B}$$

where the floating base current (*I*_B_) consists of stored holes formed through impact ionization. The *β*, which is related to retention characteristics, is used as a criterion for how well the stored holes can be maintained. The *M*, associated with the impact ionization rate, is used to determine how effectively the excess holes can be transferred to the p-base region. According to Eq. (1), it can be seen that latch-up occurs under the condition, i.e. (*M* − 1)⋅*β* = 1 where *I*_A_ momentarily diverges. The latch-up voltage is 2.9 V, which forms a positive feedback system consisting of hole generation and retention. Then, the device switches from *HRS* to *LRS*. After the latch-up, the high *I*_A_ is observed in a high voltage region for (I) *V*_AC_ ≥ 2.6 V. This is because a high electric field is formed, increasing the electric field at the *n*-*p*_1_ junction and amplifying excess holes. The stored holes in the *p*-base lower the energy barrier height, facilitating the flow of electrons to the p-base. As more electrons flow into the base region, this process continues to repeat, increasing *β*. In other words, when the *V*_AC_ corresponding to section (I) is applied after latch-up, the device shows *LRS*. Next, it can be seen that the *I*_A_ in section (II) 1.1 V ≤ *V*_AC_ < 2.6 V is significantly lower than that in section (I). This is the result of band offset engineering, where the *p*-base is designed by splitting it into *p*_1_-base (Si) and *p*_2_-base (SiGe). Moreover, a particularly notable aspect is the gentle slope of the *I*_A_ in section (II), characterized by 523 mV/dec. This means that the device has a significant margin for determining the *V*_hold_.

Figure 3c shows the energy band diagram and hole density at *V*_AC_ = 1.1 V, the voltage within section (II), providing a comprehensive analysis of the low-level *I*_A_ resulting from band offset engineering. As *V*_AC_ decreases, the number of generated holes decreases due to weak impact ionization, which is characterized by low *M* and *β* values. The generated holes are not evenly distributed over the entire *p*-base; instead, they are accumulated locally within the *p*_2_-base. This occurs because the *p*_2_-base with valence band offset has better hole retention characteristics than the *p*_1_-base. The *p*_2_-base accumulates most of the generated holes in the valence band offset region, thereby lowering the energy barrier. In comparison, the *p*_1_-base, where few holes are stored, has a high energy band level, forming a hump that prevents the inflow of electrons. Thus, this heterojunction adjusts the positive feedback system to have a low *I*_A_ by physically separating the *p*_2_-base from the *n*-*p*_1_ junction where impact ionization occurs. In addition, this positive feedback system can be strategically adjusted by modulating the hump height in terms of the doping and length of the *p*_1_-base. Figure 3d compares the quasi-static hysteresis curve when the doping of the *p*_1_-base is 5 × 10^17^ and 3 × 10^17^ cm^−3^. When the *p*_1_-base doping decreases from 5 × 10^17^ to 3 × 10^17^ cm^−3^, the *V*_hold_ decreases from 1.1 to 1.0 V, and *I*_A_ increases from 35.7 to 389.8 fA. This is because low *p*_1_-base doping reduces the hump height, promoting positive feedback even at smaller *V*_hold_. In other words, there are trade-offs. a lower hump height decreases the *V*_hold_ when the doping or length of the *p*_1_-base decrease, but this also results in an increase in the standby *I*_A_. As a result, setting the *V*_hold_ to 1.1 V can minimize standby power consumption, thereby maintaining the stored holes at a very low standby *I*_A_ of 35.7 fA.

Figure 3e shows the stored hole densities as a function of standby time, confirming the persistent hole retention characteristics when the *V*_hold_ of 1.1 V is applied. As the standby time increases, the majority of the holes stored in the *p*_1_-base decreases, while the hole density stored in the *p*_2_-base converges to 8.4 × 10^17^ cm^−3^. This result highlights the stable maintenance of stored holes even under steady-state conditions, in contrast to the case in Fig. 3a where *V*_hold_ is not applied. Figure 3f shows the program and subsequent read operations at *V*_hold_ of 1.1 V to verify the normal read operation under the ultra-low current retention condition. The standby time between them is 10 s, which is sufficient for the memory to reach a stable steady state. After the program operation, the standby current converges to an ultra-low level of 35.7 fA. Afterwards, the read current rapidly increases to a level where the switched state can be normally detected. This occurs because the positive feedback system is activated in the same direction as programming. These results indicate that our device can maintain the detectable programmed state with the ultra-low standby current level, without a requiring refresh operation. Notably, this positive feedback system does not occur when reading the *HRS*. This is because the reading voltages are insufficient to overcome the high barrier of the unprogrammed state. Therefore, our proposed device provides a non-destructive read operation. Unlike conventional DRAM devices, the stored data remains undamaged during the reading process.

## Conclusion

We have proposed SBRAM based on heterojunction bandgap engineering for high-density and low-power memory. This device can achieve high-density array configurations due to its simple vertical stacking structure. Also, nanosecond switching is achieved through a positive feedback latch-up mechanism, which involves the impact ionization effect and instantaneous charge generation. In particular, the Schottky barrier at the anode electrode enables unidirectional conduction, effectively blocking the sneak current paths. Heterojunction bandgap engineering can adjust the positive feedback system, resulting in the three different current levels determined by the *V*_hold_. The important thing to note here is that the gradual current slope ensures sufficient *V*_hold_ margin. With a minimum *V*_hold_ of 1.1 V, SBRAM can remain the programmed state at an ultra-low standby current of 35.7 fA without the refresh operation. Consequently, our proposed memory structure can be an excellent candidate for high-density and low-power capacitor-less memory solutions.

## Data Availability

The data generated and/or analyzed during the current study are not publicly available for legal/ethical reasons but are available from the corresponding author on reasonable request.
